# Cardiac electrophysiological alterations and clinical response in cardiac resynchronization therapy with a defibrillator treated patients affected by metabolic syndrome

**DOI:** 10.1097/MD.0000000000006558

**Published:** 2017-04-07

**Authors:** Celestino Sardu, Matteo Santamaria, Stefania Funaro, Cosimo Sacra, Michelangela Barbieri, Pasquale Paolisso, Raffaele Marfella, Giuseppe Paolisso, Maria Rosaria Rizzo

**Affiliations:** a Department of Medical, Surgical, Neurological, Metabolic and Aging Sciences, Second University Study of Naples, Naples; b Cardiovascular and Arrhythmias Department, Giovanni Paolo II Research and Care, Foundation, Campobasso, Italy.

**Keywords:** cardiac resynchronization therapy with a defibrillator, heart failure, metabolic syndrome

## Abstract

Metabolic syndrome (MS) is a multifactorial disease that can affect clinical outcomes in patients treated by Cardiac Resynchronization Therapy with a defibrillator (CRT-d).

Ninety-one patients received a CRT-d. According to clinical diagnosis, the study population was divided into 46 MS (cases) versus 45 no MS (controls) patients. These patients were followed by clinical, instrumental assessment, and device telemetric interrogations at follow-up. The design of the study was to evaluate the functionality of the CRT-d leads, the arrhythmic events, the CRT-d response, and the clinical outcomes at follow-up.

At follow-up, there was a statistical significant difference, comparing MS versus no MS patients regarding the sensing, pacing, and impedance thresholds of the right atrium, right ventricle, and left ventricle leads. There was a statistically significant difference in the percentage of CRT-d responders comparing MS (n = 16, 51%) versus no MS (n = 40, 77%) patients (*P* = 0.017). MS may be predictive for hospitalization for heart failure worsening (hazard ratio 0.327, 95% confidence interval 0.096–0.943, *P* = 0.044) in CRT-d patients.

MS is a complex multifactorial disease that may affect the functionality of CRT-d leads, the CRT-d response, and clinical outcomes in failing heart patients. These parameters may be detectable by follow-up monitoring.

## 1 Introduction

Obesity, hypertension, diabetes, and dyslipidemia are risk factors leading to metabolic syndrome (MS).^[1]^ MS patients have an increased risk for coronary heart disease, cardiovascular disease, and all-cause mortality.^[2–4]^ MS is related to higher percentage of cardiovascular disease progression toward heart failure (HF).^[5]^ In MS patients, HF disease progression, and its clinical stage, are related to a pro-thrombotic, and pro-inflammatory state.^[6,7]^ In HF patients, cardiac resynchronization therapy with a defibrillator (CRT-d) may improve symptoms, quality of life, New York Heart Association (NYHA) class, and clinical outcomes.^[8]^ CRT-d may prevent worse clinical events in failing heart patients.^[8]^ Moreover, CRT-d is a choice treatment for chronic HF patients, despite optimal medical treatment, with severely depressed left ventricle ejection fraction (LVEF ≤35%), and complete left bundle branch block.^[9,10]^ Obesity, hypertension, diabetes, and dyslipidemia (MS risk factors) may differently affect HF patients prognosis. In fact, MS may be associated with altered oxide reductive and inflammatory tone, which may affect ionic channels conductions properties, and altering the sympathetic tone balance.^[11]^ These alterations in channels conduction properties, and subsequently in the cardiac structure, may cause cardiac arrhythmias, by conditioning the stage of heart disease, and determining the progression to more severe forms.^[11]^ On contrary, failing heart patients affected by MS may be treated by a CRT-d.^[9,10]^ Patients treated by CRT-d have leads [right atrium, right ventricle (RV), and left ventricle (LV) leads] implanted in the heart chambers. These leads are permanently implanted, and used for sensing, pacing, and defibrillating functions. Therefore, we may speculate that, the monitoring of CRT-d leads, may give us indirect information about cardiac, and myocyte electrophysiological properties, and functions. To our knowledge, there are not clinical studies investigating electrophysiological alterations in failing heart and MS patients treated by CRT-d. In our study, we investigated the functionality of CRT-d leads (sensing, impedance, and pacing thresholds), and the electrophysiological properties of CRT-d patients comparing MS with no MS patients (cardiac electrophysiology study), by monitoring, and interrogation of CRT-d devices. In these patients, we reported hospitalization rate, arrhythmic burden [atrial fibrillation (AF), ventricular tachycardia (VT), and ventricular fibrillation (VF) events, and implantable cardioverter defibrillators (ICD) shocks], cardiac death events, all cause of deaths, stroke events, and percentage of CRT-d responders (clinical study). Our study hypothesis was that, MS may lead to alterations of cardiac electrical properties, and cardiac structure (fibrosis and fat deposition) in failing heart patients treated by CRT-d. Therefore, MS may alter CRT-d functions, conditioning CRT-d response, clinical events, and clinical outcomes in failing heart patients.

## 2 Methods

**Figure 1:**
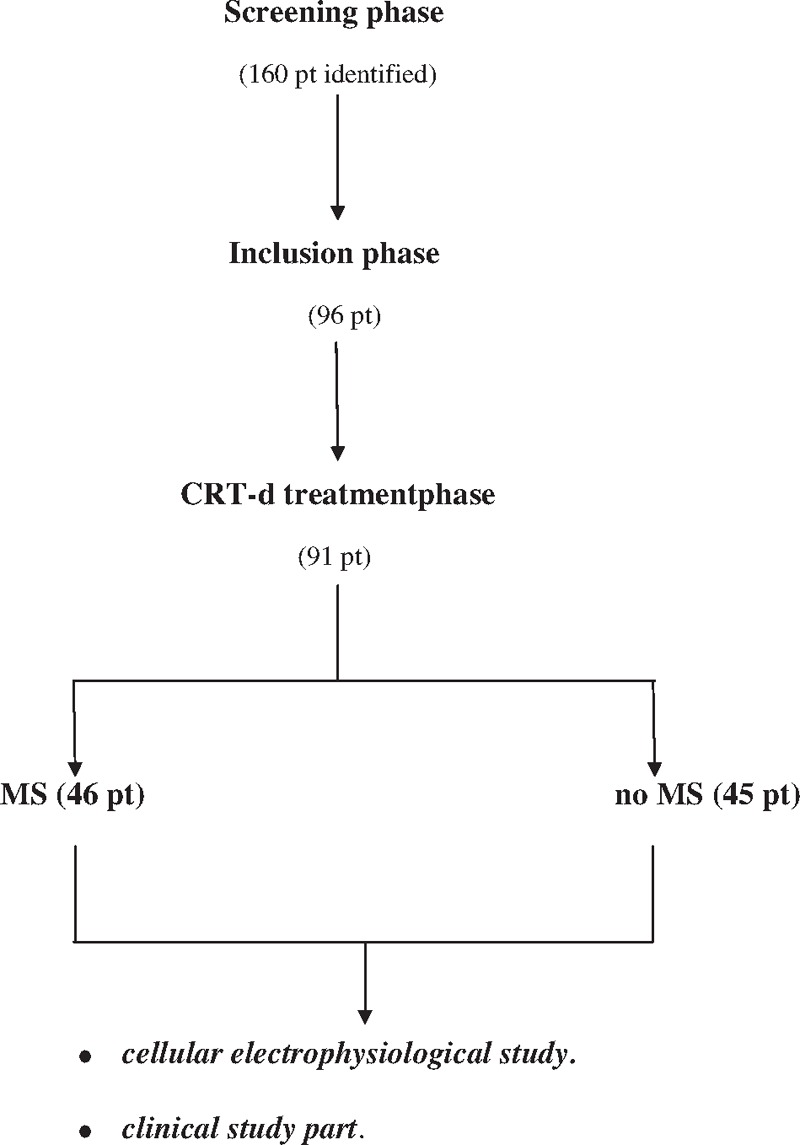
Study flow chart representations. One hundred sixty patients **[**with chronic heart failure lasting for at least 3 months, New York Heart Association (NYHA) functional class II or III, left bundle brunch block, severe LVEF reduction (LVEF <35%)] and an indication for CRT-d treatment have been identified and screened for participation in this study. Ninety-six eligible patients were included in the study. Ninety-one patients received a CRT-d treatment. These patients were divided into metabolic syndrome (MS) patients, versus no MS patients (46 vs 45 pt). After the CRT-d treatment, these patients were ambulatory monitored.

We screened 160 consecutive patients with stable chronic HF, NYHA functional class II or III, left bundle branch block, severe LVEF reduction (LVEF <35%), and an indication for CRT-d treatment according to the international guidelines.^[9,10]^ Exclusion criteria were as follows: age <18 or >75 years, ejection fraction >35%, previous ICD, CRT-d and/or pacemaker implant, absence of informed patient consent, and any condition that would make survival for 1 year unlikely. Furthermore, patients with prior cardiac surgery were excluded. Ninety-six eligible patients were included in the study, and 91 patients received a CRT-d treatment, and a traditional CRT-d ambulatory monitoring (Fig. 1). All patients were informed about the study nature, and gave their written informed and signed consent to participate in the study. Study population was divided into controls and MS patients, according to the diagnostic-specific criteria.^[1,12]^ In this population, 2 patients have refused to participate in the study, 1 has wrote down the study consent, and 2 have refused to receive a CRT-d (Fig. 1).

### 2.1 Study protocol

After enrollment, 91 patients received a CRT-d, and then divided into MS patients, and control group (46 MS vs 45 no MS patients; Fig. 1). Before interventions, baseline laboratory studies, including HbA1c, lipid panel, and fibrinogen, were determined. Follow-up was concluded at 12 months after CRT-d implant. Responders patients to a CRT-d treatment were defined by evidence of left ventricular reverse remodeling, 6 minutes-walk improvement, and Minnesota Living with Heart Failure scale improvement.^[9,10]^ Enrolled patients were followed by clinical, instrumental assessment, and device telemetric control (10 days, 6 months, and 12 months after discharge). During these visits, and device interrogations, we reported lead functionality parameters, and arrhythmic events in CRT-d recipients (cardiac electrophysiology study), and subsequently its effect in terms of clinical outcomes, as CRT response entity, and clinical events (clinical study; Fig. 1). This multicenter prospective study was conducted from September 2012 to December 2014 at Catholic University of Sacred Heart, Campobasso, Italy, at Giovanni Paolo II Research and Care Foundation, Campobasso, Italy, and at Second University Study of Naples, Italy. The study was conducted in accordance with the Declaration of Helsinki. The protocol was approved by the Ethics Committees of all participating institutions.

### 2.2 Cardiac electrophysiology study

**Figure 2:**
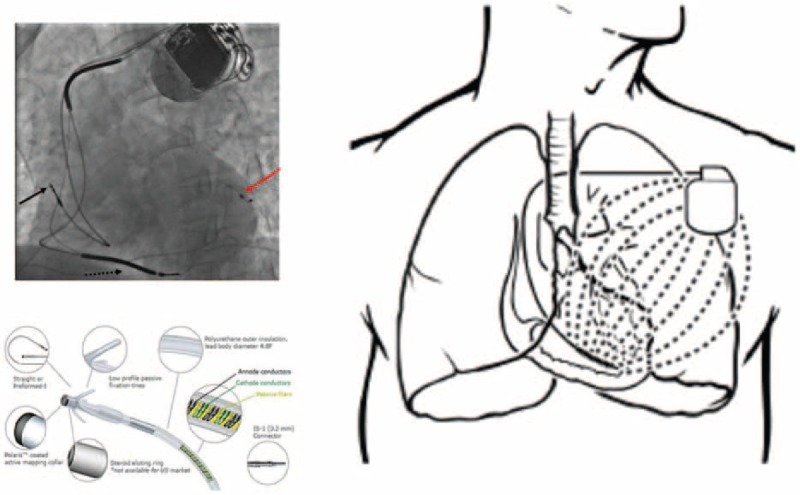
Representation of biventricular cardiac resynchronization therapy with defibrillator (CRT-d), lead structure, components and section (left upper part), and electrical impedance, pacing and sensing CRT-d field (right part). In left upper part, in a left anterior oblique radiographic view, image shows that the red arrow is indicating the left ventricular lead, the black arrow is indicating right atrial lead, while black not continuous arrow is indicating the right pacing and defibrillator lead. In left inferior image part, representation of lead structure and its components. The electrode is implanted in myocardial wall. From device interrogation, we could have information about lead sensing, impedance, and pacing thresholds. Sensing is the P and/or R wave amplitude measurement expressed in milli Volt (mV). The impedance is the measurement of a constant current sent through the tissue between the “stimulation” electrode pair (with a measurement frequency of 16 Hz, asynchronous with the cardiac cycle, therefore calculated intrathoracic impedance, expressed in Ohm). Pacing threshold in mV for milliseconds (msec) is the output lower energy to have a myocardial wall capture. These functionality parameters are related to lead position, and contact in myocardial wall, and to the lead structure integrity. In the right part of figure, representation of electrical device action field.

Right atrium, RV, and LV leads functionality parameters (sensing, impedance, and pacing thresholds) were measured as reported, and indicated by international guidelines.^[9,10]^ These parameters were P, and R-wave amplitude values (sensing thresholds), lead impedances values (impedance thresholds), and lead pacing outputs values (pacing thresholds; Fig. 2). We monitored, measured, and reported modifications of these 3 parameters in the following intervals: during implantation, at 6th month after implantation (first follow-up), and at 12th month after implantation (second follow-up), according to authors suggestions.^[13]^ The sensing thresholds values, defined as P wave and R wave sensing amplitude, were obtained from the intracardiac electrograms records, measured using a sensing configuration.^[9,10]^ The pacing thresholds, and impedance thresholds values were measured using pacing catheter configurations.^[9,10]^ To measure intrathoracic impedance (Ohm), and pacing thresholds (Volt for ms), we focused on the RV coil electrode to device case pathway configuration, and on the LV tip to LV ring configuration.^[14,15]^ A constant current was sent through the tissue between the “stimulation” electrode pair with a measurement frequency of 16 Hz, asynchronous with the cardiac cycle. The resulting voltage, 141 and therefore calculated as intrathoracic impedance, was acquired from the “measurement” electrode pair^[14,15]^ (Fig. 2). These parameters were evaluated during ambulatory follow-up visits, and device interrogations at follow-up. All CRT-d implant procedures were standardized. Right atrial catheters were all placed in right atrial appendage, and right ventricular catheters in RV apex, as indicated by anteroposterior, right anterior, and left anterior oblique views projections at radioscopic imaging. Left epicardial catheters were placed by percutaneous coronary sinus catheterization. In this case, we have chosen, by previous described radioscopic projections, a lateral and/or posterior-lateral target vessel, according to international guidelines recommendations.^[9,10]^

### 2.3 Clinical study

Hospitalization rate was reported during telephonic interviews, by hospital admissions schedules, by hospital discharge schedules, and during medical interrogation at follow-up visits. The arrhythmic burden (AF, VT and VF episodes, and ICD shocks), and the percentage of CRT-d responders were evaluated for each patients in the 2 study groups, at first (6 months after CRT-d implant), and second follow-up phase (12 months after CRT-d implant), by ambulatory device monitoring, clinical assessment, and telephonic interview. The percentage of CRT-d responders patients was evaluated by periodic clinical examinations, and echocardiography assessments.^[9,10]^ Cardiac deaths, all cause of deaths, and strokes events were evaluated during office follow-up visits 10 days after clinical discharge, and after 6th and 12th months by the treating physician, by telephonic interviews, hospital admissions, and discharge schedules. At each clinical follow-up, right atrial, right ventricular, and left ventricular leads functionality, atrial and ventricular arrhythmias, ICD shocks, and biventricular pacing percentage, were evaluated and reported for each patient. NYHA classification was reassessed, and patients graded their overall condition as unchanged or slightly, moderately, or markedly worsened, or improved since randomization by global self-assessment.^[16]^ All patients were instructed to report about devices alarms, loss of lead capture, phrenic nerve stimulation, and arrhythmias. All patients were instructed regularly to assess body weight, occurrence of dyspnea, and any clinical symptom. At each visit, patients were asked whether medical events or symptoms suggestive of cardiac arrhythmias occurred, and an ECG, and an ECG Holter monitoring, were performed to detect the presence of asymptomatic arrhythmias. Clinical evaluations included physical examination, vital signs, and review of adverse events. A fasting blood (at least 12 hours from last meal) was performed for glycaemia, lipid profile [total cholesterol (TC), triglycerides, high-density lipoprotein-cholesterol (HDL-C), and low-density lipoprotein-cholesterol (LDL-C)], and C-reactive protein (CRP) at every visit.

### 2.4 Study endpoints

As primary study endpoints, we reported the changes in the functionality of catheters parameters (sensing, impedance, and pacing thresholds), and hospitalization rate in MS as compared with no MS patients. As secondary endpoints, we assessed arrhythmic burden (AF, VT, and VF events, ICD shocks), and percentage of CRT responders comparing MS patients with no MS patients.

### 2.5 Statistical methods

All collected data were analyzed by a qualified statistician. The patients were divided before in MS group and no MS group (control group), and during follow-up visits and controls in CRT-d responders versus CRT-d nonresponders. We postulated that the number of patients with alterations in lead functionality parameters, CRT-d responders number, and secondary endpoints, was significantly different between MS patients and no MS patients. Safety analyses were performed on data from all enrolled patients. Continuous variables were presented as mean and standard deviation if normally distributed; otherwise, they were presented as median and interquartile range. Categorical variables were expressed as number and frequencies. Continuous variables were compared with an unpaired Student *t* test, and categorical variables were compared using the Chi-square test or Fisher exact test as appropriate. Predictors of the primary study endpoint were evaluated by using Cox regression models in which covariates for the adjustment were selected if associated with a *P* value ≤0.25 at univariate analysis. A stepwise method with backward elimination was used and hazard ratios (HRs) with 95% confidence intervals (CIs) were derived. We considered a 2-sided *P* value of less than 0.05 as statistically significant. The statistical analysis was performed using the SPSS software package for Windows 17.0 (SPSS Inc., Chicago, IL).

## 3 Results

Ninety-one patients treated by a CRT-d (46 MS vs 45 no MS patients) completed the study follow-up (Fig. 1). Median population age was 67 (53–75) versus 68 (54–75) years (*P* = 0.425), and male sex was 33 (72%) versus 32 (71%) (*P* = 0.986), comparing MS patients versus no MS patients (Table 1). Clinical characteristics at enrolment were similar, and balanced between the 2 groups of patients (MS vs no MS patients), a part of diabetes, overweight [described as body mass index (BMI) >30], and dyslipidemia, which were more frequents in MS patients than in no MS patients. In fact, diabetic patients were 29 (63%) versus 14 (31%), (*P* = 0.05), obese patients were 31 (67%) versus 13 (29%), (*P* = 0.02), and dyslipidemic patients were 37 (82%) versus 24 (52%) (*P* = 0.032), comparing MS with no MS patients, respectively (Table 1). In MS patients as compared with no MS patients, there was a higher percentage of ischemic cardiac disease [35 (77%) vs 25 (55%), *P* = 0.047; Table 1]. About pharmacological treatment in 2 groups, there was a statistical significant difference about lowering lipid drugs, insulin therapy, oral anti diabetic medications, and antihypertensive drugs [32 (71%) vs 23 (52%), *P* = 0.042; 15 (32%) vs 4 (9%), *P* = 0.041; 26 (58%) vs 13 (29%), *P* = 0.022; 18 (40%) vs 8 (17%), *P* = 0.043; 23 (51%) vs 15 (32%), *P* = 0.033; 34 (74%) vs 17 (38%), *P* = 0.034], comparing MS versus no MS patients, respectively (Table 1). During follow-up, primary and secondary study endpoints were reported in study population, comparing MS patients with control patients (Table 2). In a post-hoc analysis, we evaluated all different parameters revealed by clinical visits, and CRT-d devices monitoring, to differentiate CRT-d responders versus nonresponders, and to study the primary and the secondary study endpoints. Therefore, study population (MS and no MS patients) was divided into CRT-d responders versus CRT-d nonresponders, as described before by clinical characteristics, and CRT-d response during follow-up.^[9,10]^

**Table 1:**
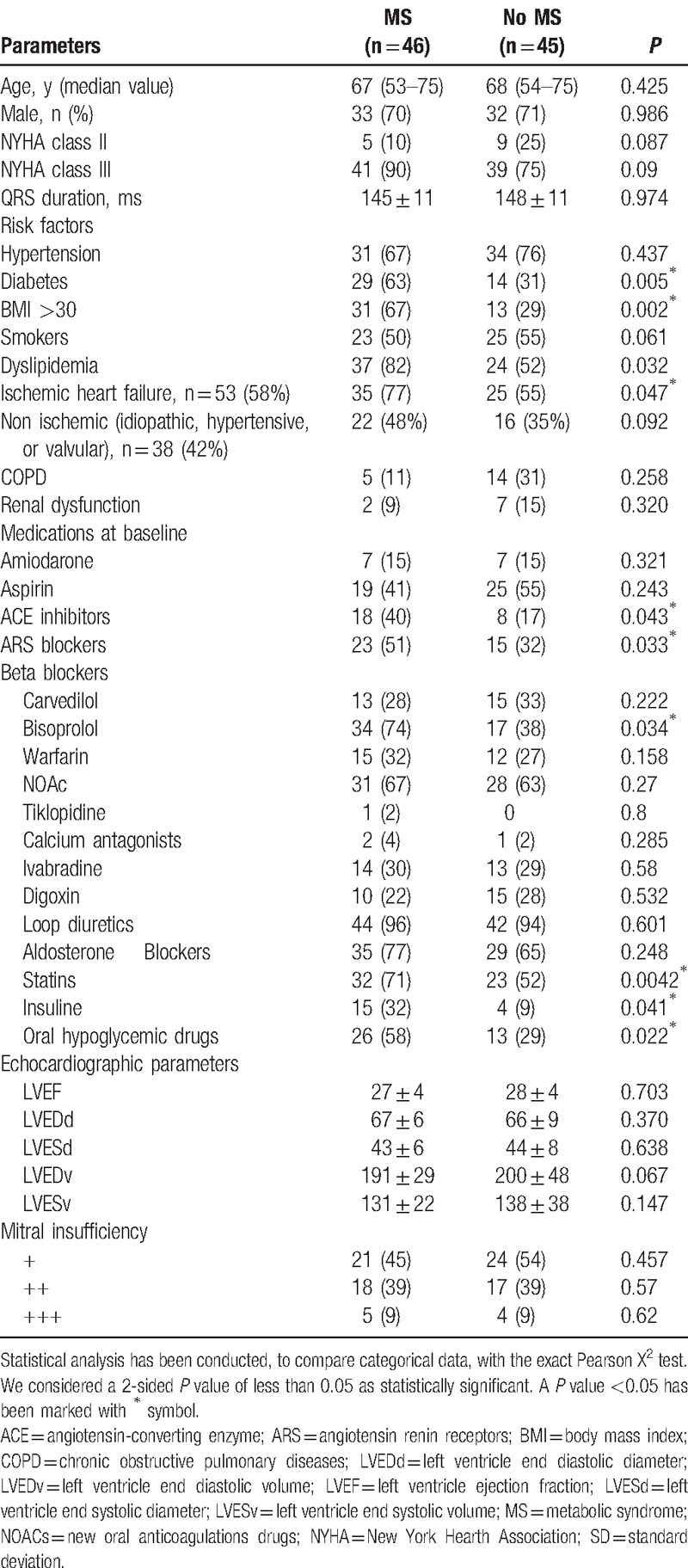
Clinical characteristics, drug therapy, and echocardiographic parameters have been reported, at baseline, comparing metabolic syndrome (MS) patients versus overall population (no MS).

**Table 2:**
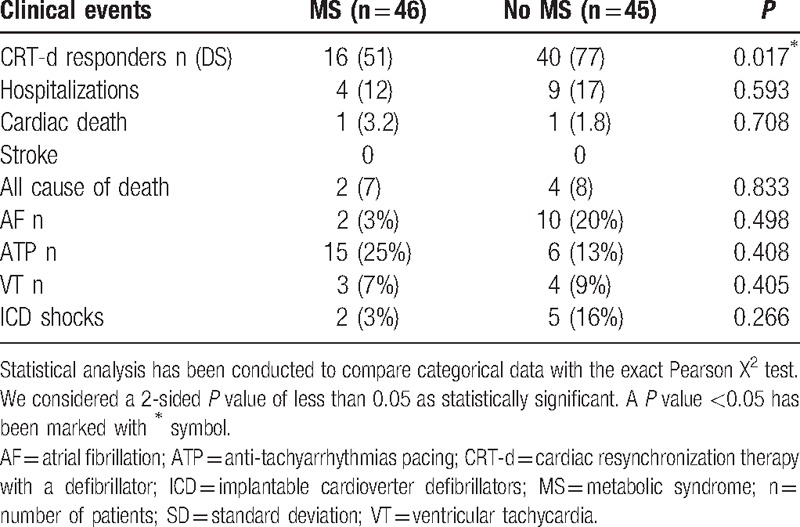
Clinical events, after cardiac resynchronization therapy with a defibrillator (CRT-d) treatment, in metabolic syndrome (MS) and no MS patients.

### 3.1 Cardiac electrophysiology study

At enrolment (CRT-d implant, phase 0), there was no significant difference about lead functionality parameters, comparing MS with no MS patients (Table 3). After first follow-up phase (phase 1), there was a significant difference comparing MS with no MS patients (MS1 vs no MS1), about LV sensing thresholds (11.8 ± 5.9 vs 13.2 ± 5.6, *P* < 0.05); right atrium impedance (422 ± 136 vs 356 ± 173, *P* < 0.05); RV impedance thresholds (433 ± 147 vs 532 ± 154, *P* < 0.05), RV shock impedance thresholds (65 ± 15 vs 72 ± 14, *P* < 0.05), RV sensing thresholds (11 ± 6 vs 13.5 ± 5, *P* < 0.05), RV pacing threshold (1 ± 0.6 vs 0.5 ± 0.3, *P* < 0.05); Table 3. At second follow-up phase (phase 2), there was a significant difference comparing MS with no MS patients about: LV impedance, pacing, and sensing thresholds (512 ± 196 vs 580 ± 169, *P* < 0.05; 2 ± 1.2 vs 1.2 ± 0.5, *P* < 0.05; 11 ± 5 vs 16 ± 5.5, *P* < 0.05); right atrial impedance, pacing, and sensing thresholds (405 ± 124 vs 326 ± 153, *P* < 0.05; 0.9 ± 0.4 vs 0.6 ± 0.25, *P* < 0.05; 1.1 ± 0.8 vs 2 ± 1.1, *P* < 0.05); RV impedance, RV shock impedance, pacing, and sensing thresholds (412 ± 132 vs 541 ± 157, *P* < 0.05; 64 ± 14 vs 72 ± 15, *P* < 0.05; 1.3 ± 0.5 vs 0.8 ± 0.3, *P* < 0.05; 12 ± 9 vs 18 ± 4, *P* < 0.05); Table 3.

**Table 3:**
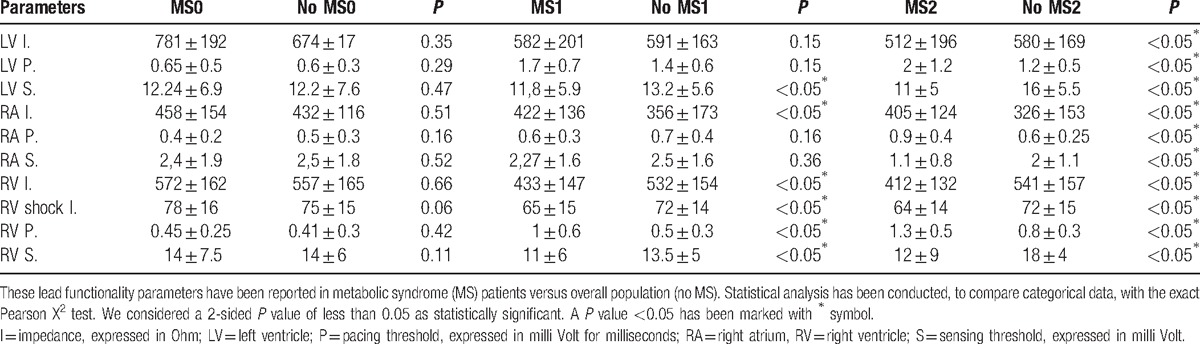
Pacemaker leads parameters measurements have been reported at baseline (0), and during first (1), and second (2) follow-up phases.

### 3.2 Clinical study

Regarding clinical events, there was a statistical significant difference about CRT-d responders percentage [16 (51%) vs 40 (77%), *P* = 0.017], comparing MS with no MS patients; Table 2. At univariate analysis, factors predicting hospitalization for HF worsening were LVEF (HR 1.130, 95% CI 1.011–1.262, *P* = 0.031), and MS (HR 0.32, 95% CI 0.123–0.832, *P* = 0.019). At multivariate analysis, MS was the only factor predicting hospitalization for HF worsening (HR 0.327, 95% CI 0.096–1.113, *P* = 0.044); Table 4.

**Table 4:**
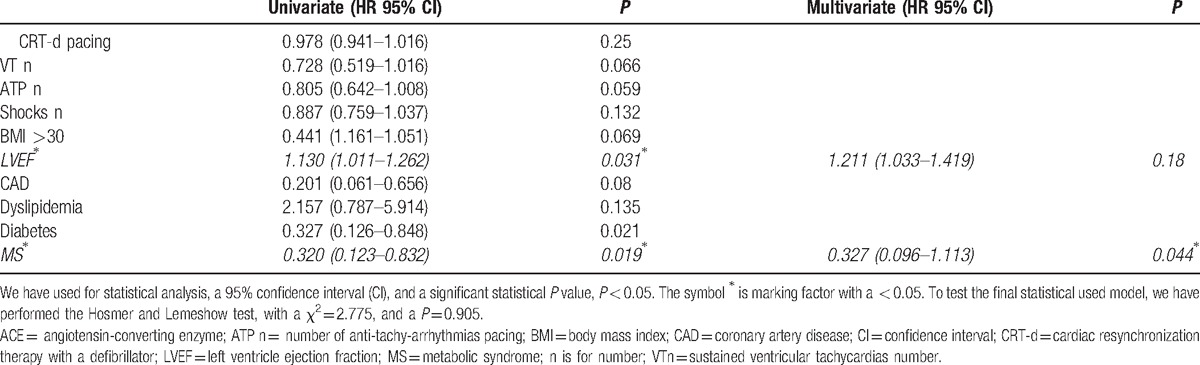
The representation of heart failure worsening univariable, and multivariable predictive factors.

## 4 Discussion

In this study, we reported a statistical significant difference about leads parameters thresholds, CRT-d response, and clinical outcomes comparing MS with no MS patients during follow-up (Table 3).

### 4.1 Leads parameters functionality and alterations

Leads parameters functionality, and their changes were collected by CRT-d devices interrogations at implant, and during follow-up. Numerous observations may explain the different mechanisms implicated in the alterations of CRT-d leads functions. As first, we may speculate that the body structure and MS risk factors may affect these parameters. In fact, MS risk factors (obesity, dyslipidemia, glucose intolerance, and hypertension) may lead to a pro-inflammatory status, and to sympathetic nervous system hyperactivity.^[12]^ These MS pro-arrhythmic conditions may condition local sensing, pacing, and impedance thresholds values in CRT-d patients. In our study, *impedance thresholds* were significantly reduced in MS patients as compared with no MS patients. Impedance thresholds, monitored by ambulatory devices interrogations, are represented by intrathoracic impedance.^[14]^ Intrathoracic impedance is due to electrical current passing across the lung, and directly to intrathoracic impedance.^[14]^ The thoracic diameters, intrathoracic fat disposition and fluids, form better conductance, causing a corresponding decrease in impedance.^[14]^ Moreover, impedance thresholds may be influenced by intrathoracic fat disposition, and thoracic structure. These alterations may affect the passage of ionic current between cellular membrane, and cellular gaps, making myocardial cells more resistant to ionic current passage. Similarly, *sensing thresholds* may be influenced by cardiac muscle structure, and composition, as muscle, fibrotic array, and fat disposition.^[13]^ In MS patients, systemic and cardiac structure alterations (body and cardiac structure composition and fat disposition) may lead to different, and lower sensing thresholds than no MS patients. The abnormal ossido-redox balance, and sympathetic tone overactivity, related to ionic channels functions and conductions, may lead to alterations of repolarizing activity. These subclinical alterations may affect local sensing values. Finally, we reported lower *pacing thresholds* in MS patients than in no MS patients. We could speculate that, MS patients as compared with no MS patients may have different cardiac electrophysiologic properties. In fact, in human and animal models, glucose homeostasis, and insulin unbalance, may lead to abnormalities in repolarizing phase.^[19]^ The “metabolic pro arrhythmic effect” may alter the calcium channel activity, leading to QT interval prolongation.^[19]^ The QT interval prolongation may trigger ventricular arrhythmias.^[19]^ In a human model, authors reported that MS may impact on clinical outcomes in arrhythmic patients.^[11]^ In fact, MS arrhythmic burden may be directly related to alterations in oxido-reductive, inflammatory tone, and sympathetic activity.^[11]^ These alterations in MS patients may lead to a pro-arrhythmic state, and this may affect the prognosis.^[11]^ In our study, at follow-up, we observed similar alterations in cardiac electrical properties by routine CRT-d devices monitoring. We may speculate that, MS patients may have higher myocardial cells refractory to artificial electrical pacing, causing a different cellular sensing, and repolarizing activity with alterations in systolic (pacing thresholds), and diastolic electrical phase (sensing thresholds).

### 4.2 CRT-d response and clinical outcomes

*Obesity* may independently be associated with worse clinical outcomes in CRT-d patients.^[20,21]^ Also, *diabetes,* another MS risk factor, may affect long-term response, and clinical outcomes in failing heart patients treated by CRT-d.^[22–24]^ The worse glycemic control, and the adverse insulin effect on cardiovascular function were previously reported, and attributed to the alteration in the relationship between the mitogenic and metabolic pathways in myocardial cells.^[24,25]^ Obesity, Diabetes, and Systemic Hypertension are strongly correlated.^[12]^ In MS patients, hypertension is linked with an increase in visceral fat.^[26]^ In fact, insulin resistance, and the sympathetic nervous system overactivity, were proposed as common mechanisms linking the other MS components.^[27]^ Sympathetic nervous system overactivity is related to urinary norepinephrine increasing, which is associated with alterations in BMI, abdominal girth, and insulin–glucose levels.^[27]^ The association between obesity, fasting insulin, insulin sensitivity, and blood pressure may be explained by phenomena related to the concomitant variation in the amount of abdominal fat.^[26]^ The systemic inflammatory state obesity induced may lead to systemic hypertension.^[12]^ In fact, a strong correlation exists between obesity, pro-inflammatory cytokines, and CRP circulating levels.^[12]^ Also, the *dyslipidemia* may affect cardiovascular system functions, conditioning the clinical prognosis.^[1]^ In fact, dyslipidemia may be related to higher percentage of ischemic cardiomyopathy, LV systolic dysfunction, and HF disease progression.^[1]^*Obesity and overweight* may influence cardiovascular diseases outcomes in failing heart patients.^[12]^ The obesity may predispose to HF through different mechanisms, as increased total blood volume, increased cardiac output, adipositas cordis, LV hypertrophy, left ventricular diastolic, and systolic dysfunction.^[12]^ Therefore, obesity may be associated with cardiac dysfunction, adipokine deregulation, and activation of the pro-fibrotic signaling pathways leading to cardiac fibrosis.^[7]^ Cardiac fibrosis is a key structural change responsible for AF.^[17]^ In fact, sustained obesity leading to pro-fibrotic Tissue Growth Factor-β1 hyperexpression may be associated with interstitial atrial fibrosis, reduced posterior left atrial endocardial voltage, and epicardial fat infiltration.^[18]^ These alterations may lead to a global bi-atrial endocardial remodeling with left atrial enlargement, conduction abnormalities, and fractionated electrograms.^[18]^ The atrial and ventricular chambers extensive fibrosis may cause conduction abnormalities, affecting cardiac electrical and anatomical properties, and leading to an increase propensity for AF.^[18]^ These alterations may condition the CRT-d response entity in MS patients. In fact, in our study, we reported a statistical significant difference about CRT-d responders percentage comparing MS with no MS patients [16 (34%) vs 35 (77%), *P* = 0.017]; Table 2. This result may be due to MS risk factors, and to the complexity of MS disease. In fact, MS risk factors, and body structure, may be factors conditioning the clinical entity of CRT-d response. The sympathetic system overactivity, and the chronic altered redox and inflammatory tone, may condition the electrical functions of cardiac cells in MS patients. At multivariate analysis, MS is a factor predicting hospitalization for HF worsening (HR 0.327, 95% CI 0.096–1.113, *P* = 0.044). Therefore, we may conclude that MS may be associated with a pro-arrhythmic status,^[11,31]^ and this may influence leads parameters functionality, and CRT-d response entity in failing heart patients. In our study, MS may condition a worse prognosis in HF CRT-d patients.

### 4.3 Study limitations

In this prospective multicenter study, there are few study limitations. As first, we examined a small percentage of MS patients treated by CRT-d, as compared with overall population. This was due to loss of patients during follow-up, and to the low adherence of patients to the study protocol as discussed in the Results session.

Second, this study was conducted at 12 months follow-up time, and this short follow-up duration may affect the long-term prognosis, and primary and secondary clinical outcomes. Third, we have to report the paucity of clinical characteristics that would provide a more accurate comparison to clinical trial subjects. We have not investigated electrical and molecular pathways in MS patients treated by CRT-d, and this may be a study limitation. In other studies, authors have investigated epigenetic effects CRT-d induced in HF patients.^[28–30]^ In fact, the electrical artificial pacing may induce epigenetic cardiac regulation in CRT-d recipients, controlling adaptive failing heart processes.^[29,30]^ In this study, we mentioned data about cardiac electrophysiology properties by CRT devices interrogations (leads functionality parameters). These parameters were examined and collected during routine devices interrogations, and we have no data by continuous devices monitoring systems.^[31]^ This may be limiting for our analysis, because continuous monitoring of CRT-d devices may impact positively on clinical outcomes.^[31]^ We have also to consider that, leads functions may be impacted by the quality of the physical interface between the lead, and the heart, and by the properties of the heart muscle. Therefore, these data cannot give us full information about myocyte electrophysiological properties, and functions. In fact, we have not conducted these experiments in animals and in vitro setting, studying indirectly cardiac electrophysiological alterations in MS versus no MS patients by ambulatory devices monitoring. An animal model could help us, in future, to test the MS cardiac electrophysiologic properties, at baseline and during electrical artificial pacing. This point needs to be discussed in future research trials.

## 5 Conclusion

In this study, we evaluated cardiac electrophysiological properties in MS patients by routinely CRT-d devices monitoring and interrogations. The simplicity of used method to monitor MS subjects implanted by CRT-d may lead us to a better knowledge in terms of MS impact in failing heart patients. CRT-d ambulatory monitoring may give us information about subclinical alterations in MS patients. These alterations may lead us to program CRT-d devices with different sensing, impedance, and pacing thresholds, and with a configuration adapted to MS patients. On the contrary, this may also represent the opportunity to explore the functionality of CRT-d leads, and indirectly the cardiac electrical properties in failing heart patients affected by MS. In this way, we may have a photography of subclinical alterations, and cardiac electrophysiological properties in failing heart subjects affected by MS. We may speculate to translate these information about electrical and functional properties of cardiac cells, into new mechanisms inside cardiac muscle systolic and diastolic electrical functions. For the first time in literature, we may have information about MS impact on heart function, and secondary on clinical outcomes in CRT-d patients. On the contrary, we may speculate that a better and intensive treatment of MS risk factors may lead to amelioration in CRT-d devices functions, to a better clinical response, and to favorable clinical outcomes in MS patients. This amelioration in CRT-d functions may reduce HF worsening in MS patients. At moment, this is a study hypothesis that needs to be investigated, and discussed in a larger clinical trial, and during a longer follow-up analysis. This study hypothesis may have in the near future an important and relevant clinical impact.
